# Subcutaneous immunoglobulins (SCIG) for chronic inflammatory demyelinating polyneuropathy (CIDP): A comprehensive systematic review of clinical studies and meta-analysis

**DOI:** 10.1007/s10072-024-07640-3

**Published:** 2024-06-28

**Authors:** Ahmed Ramzi, Subhia Maya, Nadeen Balousha, Haneen Sabet, Ahmed Samir, Merna Raafat Roshdy, Ghalia Aljarrah, Sireen Saleh, Ahmed Kertam, Ibrahim Serag, Mostafa Ramzi Shiha

**Affiliations:** 1 Faculty of Medicine, Mansoura University, Mansoura, Egypt; 2 Faculty of Medicine, Damascus University, Damascus, Syria; 3 Faculty of Pharmacy, Mansoura University, Mansoura, Egypt; 4 Faculty of Medicine, South Valley University, Qena, Egypt; 5 Faculty of Medicine, Al-Azhar University, New Damietta, Egypt; 6 Faculty of Medicine, Mansoura University, Mansoura, Egypt; 7 Faculty of Medicine, Sohag University, Sohag, Egypt; 8 Faculty of Medicine, Al-Balqa Applied University, Salt, Jordan; 9 Faculty of Medicine, Al-Quds University, East Jerusalem, Palestine; 10 Faculty of Medicine, Ain-Shams University, Cairo, Egypt; 11 Faculty of Urban and Regional Planning, Cairo University, Giza, Egypt

**Keywords:** Chronic Inflammatory Demyelinating Polyneuropathy, Subcutaneous Immunoglobulin, Systematic Review, Meta-analysis, Patient Preference, Patient-Centered Care

## Abstract

**Background:**

Chronic Inflammatory Demyelinating Polyneuropathy (CIDP) presents significant treatment challenges due to its chronic nature, varied clinical presentations, and rarity. Subcutaneous immunoglobulin (SCIG) has emerged as a maintenance therapy, offering potential advantages in administration and patient experience over the previously recognized intravenous immunoglobulin (IVIG). Methods: We included all clinical studies involving CIDP patients treated with SCIG from eleven databases up to March 2024.

**Results:**

50 clinical studies were included in the systematic review, with 22 involved in the meta-analysis. These studies offer clinical data on around 1400 CIDP patients. Almost all studies considered SCIG a maintenance therapy, with the majority of results suggesting it as a viable substitute that may offer comparable or enhanced advantages. Studies covered aspects such as efficacy, safety, quality of life, practicality, economic evaluation, and patient preference. Meta-analysis showed SCIG significantly improved muscle strength and sensory function, had fewer and milder side effects, reduced relapse rates, and received a strong preference.

**Conclusions:**

Findings suggest that SCIG for CIDP maintenance not only provides a more feasible alternative, with economic evaluations showing considerable cost reductions over time, and patient preference for SCIG being pronounced, but may also deliver comparable or superior health outcomes. Ongoing research lines on formulations, techniques, and direct comparative studies are critical to further illuminate, enhance, and expand SCIG's role in treatment.

**Supplementary Information:**

The online version contains supplementary material available at 10.1007/s10072-024-07640-3.

## Introduction

Chronic Inflammatory Demyelinating Polyneuropathy (CIDP) remains a compound challenge in neurology, due to its nature, spectrum, and rarity. It is caused by a long term immune-mediated demyelinating pathology of peripheral nerves, occurring as rarely as 1 per 100 thousand and presenting with multiple types and disease courses, with the typical and most prevalent form of CIDP involving deficiency in both motor and sensory limb functions and showing symmetry in both right and left, upper and lower, distal and proximal limb regions [1].

However, this condition can indeed be treatable, with treatment aiming to achieve and maintain significant improvement in overall function, symptoms, and quality of life, or even an end goal for some of being stable and off treatment in the long term [2].

CIDP was defined in the 1970s, its reported management with immunoglobulins began with Intravenous Immunoglobulin (IVIG) in the 1980s, Subcutaneous Immunoglobulin (SCIG) in the late 2000s and 2010s, while their official approvals came in the late 2000s for IVIG and late 2010s for SCIG [3–5].

As earlier treatments for CIDP were limited and less effective, the adoption of IVIG and subsequently SCIG significantly improved patient outcomes, representing a significant advancement in the management of this autoimmune disorder [6, 7].

The official approvals of SCIGs for CIDP maintenance from the late 2010s forward have expanded treatment options for CIDP patients; however, there is a need to further investigate their comparative benefits and tolerability both objectively and from the patient perspective [7–9].

This study intended to assess the efficacy, safety, and convenience of SCIG for CIDP maintenance treatment, using most reported data from most of the available and suited studies retrieved from diverse Databases funneled, condensed, and synthesized through the extensive and organized presentation and analyses, including but not limited to the analysis of overall function, muscle strength, sensory function, serum IgG levels, quality of life, relapse rate, adverse events, patient treatment preferences, and economic implications and considering most reported outcome-measures for the aforementioned outcomes, hence the use of the adjective "comprehensive" in the title.

## Methods

We observed the guidelines suggested by the PRISMA statement in conducting this systematic review and meta-analysis. The protocol was registered with PROSPERO, International Prospective Register of Systematic Reviews (ID: CRD42024521670).

### Eligibility criteria for included studies

For studies included in the systematic review, we considered the following criteria:

- 1) **Study design:** All clinical study designs: interventional RCTs and other clinical trials, observational prospective, retrospective or cross-sectional studies, case reports, and case series. Additionally, cost studies were included.
- 2) **Participants:** Patients with Chronic Inflammatory Demyelinating Polyneuropathy (CIDP), regardless of age, gender, severity of disease, type, or comorbidities
- 3) **Interventions:** subcutaneous immunoglobulins (SCIG) in any dosage, administration method, or regimen, either alone or in combination with other treatments.
- 4) **Comparator:** IVIG, different dose, administration method, or regimen of SCIG, placebo, no treatment, standard care, or other pharmacological interventions.
- 5) **Outcomes:** The main outcomes will pertain to the effectiveness and safety of SCIG in CIDP, using various relevant indicators, functional scores, and adverse events, additionally, cost-effectiveness, practicality, and patient-reported outcomes associated with SCIG for the treatment of CIDP.

### Excluded were

1) non-human research studies (laboratory animals, isolated cells, in-vitro), 2) Studies with IVIG alone, or any other interventions not accompanied with or compared to SCIG, 3) Secondary research studies (reviews, systematic reviews, meta-analysis), 4) Non-empirical studies (opinion pieces and hypotheses), and 5) non-peer-reviewed (Book chapters, editorials, and conference abstracts)

The aforementioned rules were of course applied to include the articles in the Systematic review first, but then regarding the meta-analysis case reports, case series, cost studies, and any study that did not assess at least one of the desired outcomes were not involved in the meta-analysis.

### Information sources and search strategy

The following databases were searched for publications that were available till 1 March 2024, then the search was updated on 30 March: PubMed/Medline, Web of Science, ScienceDirect, SCOPUS, CLINICALTRIALSGOV, CENTRAL/COCHRANE, ICTRP, Google Scholar, LILACS, Europe PMC, and SpringerLink.

Data sources were searched using the following query: ("chronic inflammatory demyelinating polyneuropathy" OR "CIDP" OR "chronic inflammatory demyelinating polyradiculoneuropathy") AND ("subcutaneous immunoglobulin" OR "SCIG" OR "HyQvia" OR "Hizentra" OR "IgPro20" OR "subcutaneous immunoglobulins"). (More details in the supplementary material).

### Study selection

After removing duplicates, two reviewers screened titles and abstracts in a blinded fashion, and studies that met the exclusion criteria were removed. Afterward, full-text screening was performed blindly by two reviewers to assess their eligibility for SR and MA. Any controversies were resolved through discussion or consultation with a third researcher if consensus was not reached.

### Data extraction

Two researchers independently extracted the data using a standardized online extraction form for all included studies in the systematic review, except case and cost studies which required 2 other suitable specialized data extraction forms.

According to the standardized data extraction form, the extracted data initially included the following:

1) study ID: author, year, 2) study design, 3) participants: Number, Age in years, Males: n (%), Disease Duration & Age at first diagnosis of CIDP, Baseline disease measures, Inclusion and Exclusion Criteria,4) Intervention: (SCIG), brand name, regimen, etc., Type of treatment (induction, maintenance, or both), 5) Comparator: Detailed as intervention if applicable. If the comparison is to baseline, we mentioned that. 6) Outcomes: Primary Outcome(s), Primary outcome measures, Secondary Outcome(s), Secondary outcome measures, 7) Follow-up, 8) Adverse Events 9) Comorbidities 10) Concise summary of main aims/objectives/study question, 11) Concise summary of main findings and conclusions, 12) settings: funding, settings/Places where interventions took place, country, conflict of interest.

In the context of meta-analysis, a systematic approach was employed to extract detailed outcome measures from the included studies. This involved standardizing treatment types and dosages to be uniformly expressed as (maintenance: previously stable participants on IVIg or SCIg, induction: treatment-naïve participants, or both), and g/kg/m, respectively. To deal with missing data, appropriate statistical analysis was conducted when only raw data was accessible using Jamovi software. Also, for data presented only in the form of bar plots, the WebPlotDigitizer tool was utilized to extract the values. Additionally, the effect measure was reported as mean and standard deviation (SD). In cases where outcome measures were presented in a different format, we calculated the mean and SD using the Meta Converter conversion tool if applicable.

### Measures of treatment effect

The following outcome measurements were used in the meta-analysis:

- **For relapse rate**, the proportion of patients who had a CIDP relapse during the subcutaneous treatment period excluding those who withdrew for any reason other than relapse. CIDP relapse was defined as clinical deterioration that required IVIg rescue treatment.
- **For overall function and disability assessment**, Rasch-built Overall Disability Scale R-ODS, Overall Disability Sum Score ODDSs, Inflammatory Neuropathy Cause and Treatment disability score INCATd, and INCAT sensory sum for overall sensory functions assessment.
- **For muscle strength assessment**, the Medical Research Council sum score MRC and Grip strength GS (measured by Martin vigorimeter or JAMAR dynamometer)
- **For functional Mobility assessment**, nine-Hole-Peg Test 9-HPT and timed meter walk.
- **For patient reported outcomes**, 1) patients' health-related quality of life was evaluated by the EuroQol 5 Dimension 5 level EQ5D5L index value and life quality index scale LQI, 2) health status by visual analog scale EQ-VAS, and 3) patient preference of SCIG or IVIG treatment.
- **For safety assessmen**t, adverse events AE frequency including: local-infusion reactions, headache, severe AE, and AE leading to withdrawal.
- **serum IgG levels**.

### Quality assessment or risk of *bias* for included studies

Two researchers independently assessed the quality of each included study in accordance with tools specific to the study designs as follows:

- Assessing the Risk of Bias for clinical trials:The risk of bias was assessed using the Cochrane Collaboration's tool for randomized trials ROB2 and the ROBINS-I tool for non-randomized studies. Assessments will be made at the study level, focusing on domains such as the randomization process, deviations from intended interventions, missing outcome data, measurement of the outcome, and selection of the reported result.
- Characteristics and Criteria for Assessment: Key characteristics assessed will include the method of randomization, treatment allocation concealment, blinding of participants and personnel, completeness of outcome data, selective reporting, and other biases. Each domain will be judged as 'low risk,' 'high risk,' or 'some concerns.'
- Assessing quality for other study designs: CASP Checklists (Critical Appraisal Skills Programme Checklists) were employed for cohort studies and cost studies.

### Strategy for data syntheses

Data synthesis combines meta-analysis for quantifiable data, with narrative synthesis providing context and insights for all findings.

**Narrative Qualitative Synthesis:** for studies that were unsuitable for meta-analysis, a narrative synthesis was provided. This synthesis offers a comprehensive overview by summarizing findings through:

- Thematic Analysis: Identifying recurring themes, concepts, and patterns by coding and clustering qualitative data.
- Synthesis of Findings: Creating a cohesive narrative that highlights key insights, trends, and implications from the collected evidence.
- Contextualization and Interpretation: Placing the synthesized narrative within broader conceptual frameworks to interpret the relevance of findings to both practice and future research.

**Meta-Analysis and Statistical Quantitative Syntheses**: for data suitable for quantitative analysis, the following was employed:

We used mainly RevMan 5.3 software and in a few instances OpenMeta-analyst. Changes in continuous variables were pooled as a standardized mean difference (SMD) or mean differences (MD), depending on the specific analysis. Both fixed effects and random effects models were adopted as appropriate. Fixed effects model, which is characterized by a wider standard error, a larger weight to smaller studies, and a wider confidence interval, was used for low variability.

Visual assessment of the forest plots was used to determine heterogeneity, and the I^2^ and χ^2^ (χ2) tests to measure it. The presence of notable heterogeneity was investigated using the χ2 test, and if heterogeneity was found, it was quantified using the I^2^ test. The Cochrane Handbook's guidelines for meta-analysis were followed when interpreting the I^2^ test (0-40% = may not be significant, 30-60% = may represent moderate heterogeneity, 50-90% = may represent substantial heterogeneity, and 75-100% = significant heterogeneity).

### Analysis of subgroups

Subgroup analyses were conducted to assess the differential effects of SCIG based on dosage for MRC, walk test type, and adverse events in comparison to placebo and IVIG. Dosage subgroups were categorized as low, medium, and high. Based on our standardization for dosages to be expressed as g/kg/month, we classified them as follows:

- Low dose group: 0.87, 0.87, 1, 1, 1, 1.1, 1.15
- Medium dose group: 1.24, 1.3, 1.3, 1.3, 1.3, 1.44, 1.5, 1.6
- High dose group: 1.7, 1.74, 1.74, 1.74, 1.74, 1.74, 1.74

For the Timed Meter Walk Test, the 10-Meter Walk Test (10-MWT), and 40-Meter Walk Test (40-MWT) scores were analyzed separately, and as a total. Adverse events were evaluated by comparing the occurrence rates in SCIG versus placebo and SCIG versus IVIG groups.

## Results

### Description of studies

#### Results of the search

On March 1st, 2024, 2846 studies were initially identified then reduced to 1166 by search settings through the following 11 electronic databases:

Web of Science (n = 181), Scopus (n = 85), PubMed (n = 61), Google Scholar (n = 326), SpringerLink (n = 141), CENTRAL (n = 85), Europe PMC (n = 189), LILACS (n = 1), ScienceDirect (n = 64), ClinicalTrials.gov (n = 15), and ICTRP (n = 18).

**Fig. 1:**
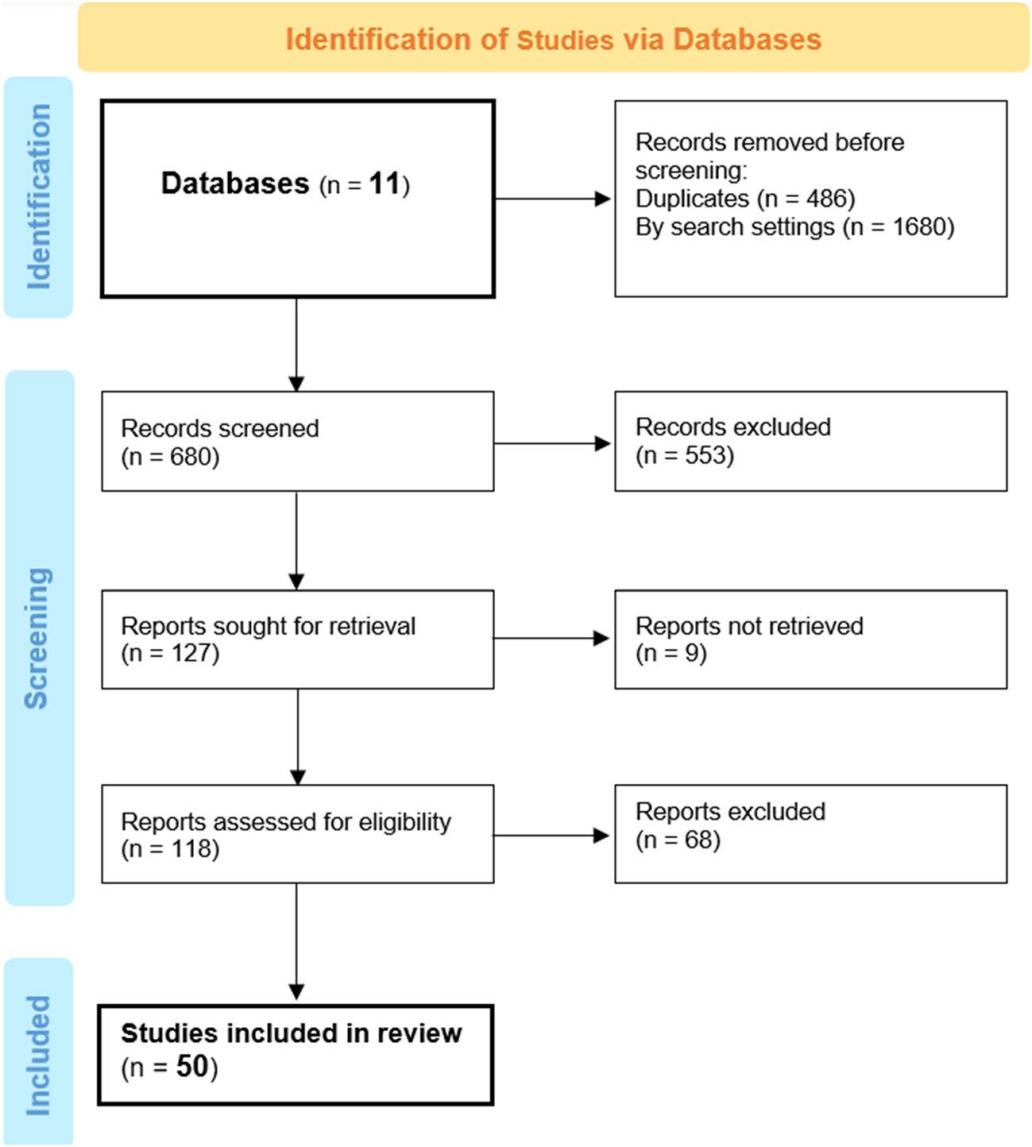
Study selection flow diagram

The search was updated on March 30th but yielded no extra studies for inclusion. Eventually, 50 clinical studies were included for the systematic review, and 22 of them were involved in the meta-analysis. Excluded studies were of course those that did not meet the inclusion criteria specified and detailed in the Methods section. The flow of that process is visually summarized as a flowchart. (Fig. 1).

### Included studies

Included studies offer clinical data on around 1400 CIDP patients, mostly from western Europe and the US, which might represent near 10% of their entire CIDP populations. The 50 studies are 6 RCTs, 10 non-randomized clinical trials, 10 observational cohort studies, 18 case series and reports, 5 cost studies and one cross-sectional.

Tabular presentations for included studies are sufficiently provided in the supplementary material and for convenience, we included only one compact table (Table 1) for summarizing study characteristics and it contains only clinical trials.

**Table 1:** Summary of characteristics of the included clinical trials

Study ID	Design	Sample size	Age (mean)	Intervention (SCIG)	Comparator	Period months	Concise Summary of conclusion
Bril 2023 (ADVANCE- CIDP1)	Phase 3 Double Blinded RCT	Total: 132 Placebo: 70 SCIG: 62	53.9 55.0	fSCIG (HyQvia) *Dose*: 1.1 g/kg/month *Duration*: 6 months	Placebo	6	fSCIG 10% resulted in > 20% reduction in relapse rate VS placebo. Although adverse events were more frequent with fSCIG 10% than placebo, severe and serious AEs were less common
Hansen 2023	Non- randomized Controlled trial	Total: 104 SCIG: 12 Control: 55	66 57	*Dose*: 0.33 g/kg/week *Duration*: 18 days	IVIg Healthy control	0.6	IVIg resulted in improvement of NET and clinical functional tests, but no correlation was found between them. SCIG didn't induce a change in neither NET nor clinical function
Markvardsen 2023	RCT	Total: 55 Relapse: 35 Remission: 20	61.25 57.25	Gammanorm or Hizentra *Dose*: was reduced stepwise -Relapse group: 0.37 g/kg/w -Remission group: 0.28 g/kg/w Duration: 60 weeks	-Frequent vs Less frequent evaluation -Relapse vs Remission	13.8	In stable CIDP patients, SCIG could be completely tapered off in 36% of the patients and only in 10% of these patients relapse occurred during the following two years. More frequent evaluation was not superior to detect deterioration
Vu 2021	open-label single arm trial	15	54.5	Hizentra *Dose*: 0.35 g/kg/week *Duration*: 24 weeks	Baseline	5.5	Out of 15 patients switched from IVIg to SCIG, 3 (20%) met the primary endpoint and one (7%) met the secondary endpoint. The switch to SCIg was associated with maintenance of efficacy and enhanced QoL
Cocito 2020	Randomized unblinded Crossover trial	Total: 10 Pump: 5 MPT: 5	48.3	SCIG by pump Dose: 0.25 g/kg/week Duration: 8 months	SCIg by MPT	8	MPT and pump infusion were similarly effective MPT slightly increased plasma IgG levels enhanced QoL
van Schaik 2019 (Extension)	Open-label Extension trial	82	57.6	Hizentra *Dose:* 0.2 or 0.4 g/kg/week *Duration*: 48 weeks	Baseline	11	IgPro20 provided long-term benefits at both 0.4 and 0.2 g/kg weekly doses with lower relapse rates on the higher dose, and most adverse events were mild or moderate with no serious adverse events
Cirillo 2018	Open-label single arm trial	16	61.4	Hizentra *Dose*: 0.4 g/kg/week *Duration*: 24 months	Baseline	24	SCIG resulted in a significant and long-term improvement of clinical and neurophysiological parameters
van Schaick 2018 (PATH study)	Phase 3 Double Blinded RCT	Total: 172 Placebo: 57 Low-dose SCIG: 57 High-dose SCIG: 58	56.73 58.63 56.93	Hizentra *Dose*: 0.2 or 0.4 g/kg/week *Duration*: 24 weeks	-Each dose vs placebo -High-dose vs low-dose	5.5	The primary endpoint occurred more often in the placebo group than in both SCIg groups. Both doses of SCIg were efficacious and well tolerated, suggesting that SCIg can be used as a maintenance treatment for CIDP, and over half preferred SCIg to their previous IVIg
Christiansen 2017	Open-label single arm trial	Total: 17; 14 CIDP	57.75	Gammanorm *Dose:* 0.34 g/kg/week *Duration:* 12 weeks	IVIG Dose: 1:1 to SCIG	4.6	Upon switching from IVIG to SCIG, fluctuation of muscle strength was unchanged, whereas performance fluctuations were diminished
Cocito 2017	Open-label single arm trial	7	63.17	Hizentra *Dose:* 0.25 g/kg/week *Duration:* 6 months	SCIg “bolus” regimen	6	SCIg “bolus” could be an effective and well-tolerated option for CIDP maintenance therapy
Markvardsen 2017	non-randomized Cross over Controlled trial	18	61.4	Aerobic exercise + SCIG *Dose:* 0.31 g/kg/week *Duration:* 25.4 months	Resistance exercise + SCIG	5.5	Aerobic exercise training and resistance exercise training improved fitness and strength in CIDP patients, whereas fatigue and QoL did not improve
Markvardsen 2016	Open-label trial	IVIG to SCIG:17; 12 CIDP SCIG to SCIG:13; 10 CIDP	57.75 57.25	IVIG to SCIG: Gammanorm *Dose:* 0.29 g/kg/week *Duration:* 12 weeks SCIG to SCIG: Gammanorm *Dose*: 0.27 g/kg/week *Duration*: 20 weeks	IVIG Subcuvia or Hizentra	4.6	The transition from IVIG to SCIG resulted in a slight increase in Hb levels and an enhancement of hemolytic activity. Hemolytic variable changes did not significantly differ after switching from one SCIG type to another
Markvardsen 2016	Randomized, single-blind, Cross-over trial	Total: 20 SCIG-IVIG:10 IVIG-SCIG:10	56.7 52.3	Hizentra *Dose:* 0.4 g/kg/week *Duration:* 5 weeks	IVIG Dose: 2 g/kg/day	4.6	SCIG and IVIG showed similar efficacy, but with earlier maximum improvement after IVIG than after SCIG treatment
Markvardsen 2014	Open-label single arm trial	17	55	Subcuvia *Dose: 0*.34 g/kg/week *Duration:* 12 months	Baseline	12	SCIG resulted in improvement of cIKS and MRC, whereas other parameters remained unchanged. SCIG could preserve muscle strength and disability for 1 year
Markvardsen 2013	double-blind placebo-control RCT	Total: 30 SCIG: 15 Placebo: 15	53.4 61.4	Subcuvia *Dose*: 0.31 g/kg/week *Duration*: 12 weeks	Placebo	2.76	SCIG resulted in significant improvement of muscle strength,disability and functional mobility. SCIG was well tolerated and preferred over IVIG by 70% of patients No systemic side effects were reported

A Compact List of brief IDs (author year) for the included studies:

Bril 2023[10], Hansen 2023 [11], Markvardsen 2023 [12], Svacina 2023 [13], Alonge 2022 [14], Ricciardi 2022 [15], Gingele 2021 [16], Kapoor 2021 [17], Murphy 2021 [18], Vu 2021 [19], Cocito 2020 [20], Gentile 2020 [21], Ryltoft 2020 [22], Cirillo 2019 [23], van Schaik 2019 [24], Cirillo 2018 [25], van Schaick 2018 [26], Christiansen 2017 [27], Cocito 2017 [28], Markvardsen 2017 [29], Markvardsen 2016 [30], Markvardsen 2016 [31], Markvardsen 2016 [32], Markvardsen 2015 [33], Cocito 2014 [34], Markvardsen 2014 [35], Markvardsen 2013 [36].

Adrichem 2017 [37], Hadden 2015 [38], Yoon 2015 [39], Lee 2008 [5], Hiya 2022 [40], Matsubayashi 2022 [41], Santilli 2021 [42], Alsolaihim 2020 [43], Marastoni 2020 [44], Cianci 2019 [45], Katzberg 2019 [46], Vacchiano 2019 [47], Rosso 2018 [48], Assenza 2016 [49], Debs 2016 [50], Nogues 2016 [51], Rosso 2014 [52], Bayas 2013 [53].

Mallick 2023 [54], Piscitelli 2021 [55], Lepage 2020 [56], Perraudin 2020 [57], Lazzaro 2014 [58].

**Fig. 2:**
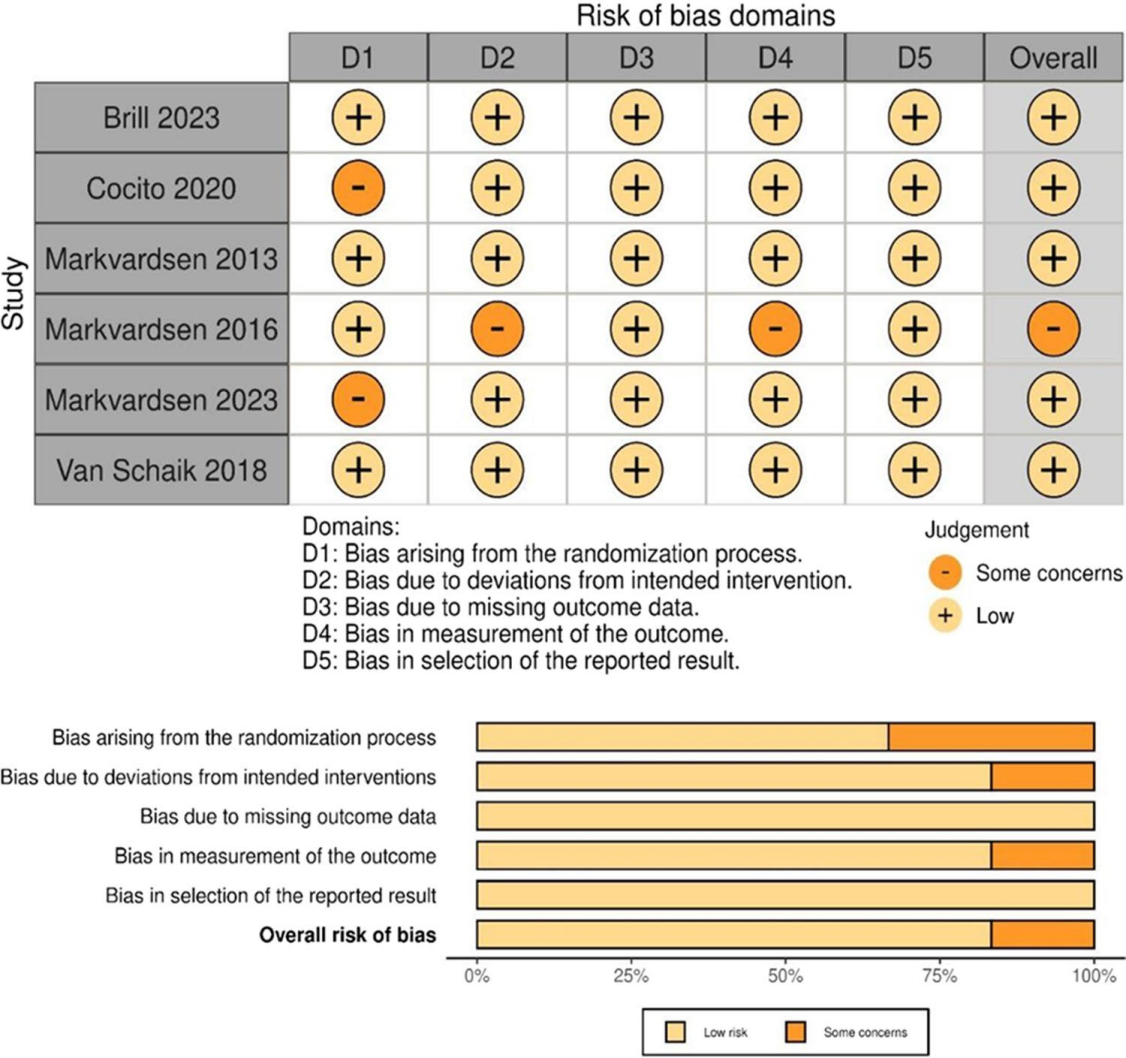
Risk of Bias Domains and Judgment for the included RCTs, using ROB2 tool

Quality or risk of bias assessments for included studies are provided in the supplementary material. Here, we offer a visual representation regarding RCTs by ROB2 depicted as (Fig. 2).

### Narrative summary of patterns and findings across studies

Considering the opportunity presented by the very large pooled sample size, we calculated the average age at disease onset and the male percentage across studies, resulting in an average age of 52 years and males being affected twice as frequently as females, which is consistent with the consensus.

Almost all studies considered SCIG to be a maintenance therapy in their context. Our analysis, though comprising multiple direct comparisons, fundamentally offers broad indirect comparisons between SCIG and IVIG, predicated on the reported facts that most patients had been previously treated with IVIG before their inclusion in the studies. Therefore, the initial disease metrics practically mirror the outcomes of IVIG treatment.

Given that SCIG is positioned as a maintenance therapy, the primary goal was to reduce relapse rates and sustain or enhance neuromuscular functions and overall ease and quality of life, with the majority of results across the various studies suggesting that SCIG not only emerges as a viable substitute but may also yield comparable or enhanced advantages in specific clinical parameters and the notable inclination towards SCIG among patients, satisfying that aim.

The majority of SCIG was Hizentra, with its manufacturer CSL Behring serving as a funding provider for many of the studies, which could partially be attributed to the high cost of human immunoglobulins in medicine. The average dose across studies and different brands was about 1.3 g/kg/month, which is equivalent to the guideline-recommended maintenance dose for CIDP using IVIG at approximately 1.44 g/kg/month [59]​. SCIG doses in the studies were generally equivalent to previous IVIG doses and fall within the recommended range for maintenance therapy. Most SCIG doses align with IVIG maintenance guidelines, indicating appropriate dosing adjustments when transitioning from IVIG to SCIG. A detailed sheet for these comparisons is provided in the supplementary material.

SCIG treatments, including fSCIG (HyQvia), Gammanorm, subcuvia and Hizentra (Igpro20), have demonstrated effectiveness in reducing relapse rates and maintaining clinical stability in CIDP patients. The findings suggest that continuous, stable dosing is crucial for maintaining remission, with some patients even able to taper off SCIG treatments without triggering relapse. This indicates potential for long-term disease management strategies that emphasize personalized dosing regimens to prevent disease progression and reduce treatment dependency.

The variation in patient responses highlights the necessity of personalized treatment plans, informed by factors like disease severity and prior treatment responses. A nuanced approach to patient monitoring is vital, as it balances regular assessments to detect potential relapse with minimizing treatment burden. Innovations in treatment administration, particularly the shift towards home-based and patient-administered treatments, underscore the movement towards enhancing treatment adherence and patient satisfaction.

A shift towards patient-centered research is evident, with an increasing focus on patient-reported outcomes alongside traditional clinical measures. This reflects a broader healthcare trend towards prioritizing patient experiences of health and treatment satisfaction.

Case studies are 4 case series, 5 reports of 2 cases each, and 9 reports of single cases.

In these studies, the general patterns identified from the larger study designs largely still hold true. Even though documenting unusual reactions and adverse events is one of the roles of case reports, regardless of how seemingly not generalizable they may be, the reported adverse effects were mostly local and transient, and more tolerable compared to those associated with IVIG.

Across multiple cases, there's a significant move from Intravenous Immunoglobulin (IVIg) to SCIg due to its convenience and reduced systemic side effects.

Several note that SCIg maintains functional stability in CIDP patients with fewer fluctuations in symptoms compared to IVIg, highlighting SCIg's role in long-term management.

Some analyses discuss the economic implications of switching to SCIg, alongside improvements in quality of life. These discussions reflect a broader consideration of treatment impact beyond immediate clinical outcomes.

With SCIg, there is an emphasis on managing local injection site reactions, suggesting a need for careful patient monitoring and support. Strategies include site rotation, managing infusion rates, and adjusting SCIg dosing based on patient feedback and symptom management, and focusing on patient training for SCIg self-administration for injection and monitoring treatment response. There's an acknowledgment of the need for better tools to monitor treatment efficacy, particularly for patients managing their therapy with self-administration at home. These could include both physical symptom trackers and digital health applications.

Apropos of self-administration, Murphy et al. (2021) conducted a retrospective analysis of 310 CIDP patients transitioning from IVIG to SCIG, dedicated to this topic [18]. It examined the efficacy of the Specialty Pharmacy Nurse Network's training program, delivered in patients' homes, aimed at achieving independent management of SCIG therapy. The majority of patients needed between three and four sessions to finish, achieving a high training completion rate of 90%. Transitioning to SCIG was not only beneficial for enhancing patient autonomy and convenience but also resulted in a reduction of systemic adverse events when compared to IVIG. However, there was a minor proportion of the cohort that discontinued treatment due to adverse events, highlighting the importance of monitoring and support during the early stages of transition.

Cost studies are all relatively recent, indicating high relevancy and the avant-garde nature of the subject matter, driven by the emerging interests.

A suite of specialized studies spanning from 2014 to 2023 offers an economic and clinical evaluation of SCIG versus IVIG treatments for CIDP across various healthcare systems. These studies collectively could enhance the understanding of the budget impact, cost-effectiveness, and patient quality of life implications associated with these treatment modalities.

Mallick 2023 (USA): This budget impact model study within the US healthcare system forecasts significant savings with SCIG over IVIG, emphasizing reduced drug and non-drug costs, modeling future impacts based on current data and assumptions about treatment costs and patient outcomes. It underscores SCIG's benefits, such as self-administration, fewer adverse events, and less patient burden.

Piscitelli 2021 (Italy): Conducted through a retrospective analysis combined with a budget impact analysis, this study is an outlier that reports higher costs for SCIG compared to IVIG. Despite these higher costs, SCIG demonstrated significant safety benefits and enhancements in patient quality of life.

Perraudin 2020 (Switzerland): It Integrates model-based cost-minimization analysis with both retrospective patient data and prospective cost estimations. It suggests SCIG as a more cost-effective alternative from a societal perspective, primarily due to lower total costs for health insurance and other payers.

Lepage 2020 (France): From the French Social Security perspective, this budget impact model predicts a 10% decrease in total annual costs per patient when switching from IVIG to SCIG over five years. It promotes SCIG as a cost-saving option that could substantially reduce hospital visits and overall treatment expenses.

Lazzaro 2014 (Italy): Conducting a cost-minimization analysis, it supports the economic benefits of SCIG over IVIG from a societal perspective, highlighting minimal annual savings favoring SCIG.

Across these diverse healthcare contexts, SCIG emerges as a generally more cost-effective treatment compared to IVIG, mainly due to its potential for reducing overall healthcare costs, including drug and non-drug related expenses. However, the Piscitelli 2021 study presents an important counterpoint by highlighting a scenario where SCIG was more expensive, which emphasizes the variability in cost-effectiveness depending on specific national healthcare system prices and patient management practices.

The studies underline similar clinical outcomes between SCIG and IVIG, suggesting that treatment decisions may be influenced more by economic considerations and patient preferences, particularly regarding quality of life. SCIG’s advantages in terms of self-administration and reduced adverse events are consistently recognized as factors that could lead to improved patient satisfaction and adherence, showing a general trend towards the cost-effectiveness and patient-centered benefits of SCIG balancing clinical efficacy with economic and quality of life considerations, especially when considering long-term treatment. 

### Meta-analysis

Meta-analysis showed SCIG significantly improved muscle strength and sensory function, had fewer and milder side effects, reduced relapse rates, and received a strong preference. These results are illustrated in Figures 3, 4, 5, 6, 7, 8, 9, and 10.

**Fig. 3:**
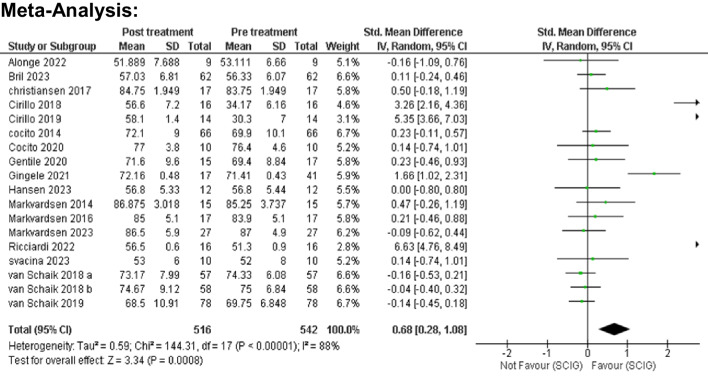
Muscle Strength (Medical Research Council Scale, MRC): Analysis incorporating data from 18 studies with a combined participant pool of 542 individuals demonstrated a significant improvement in muscle strength post-SCIG treatment. The pooled standardized mean difference (SMD) in MRC scores was 0.68 points (95% CI: 0.28 to 1.08), with statistically significant enhancement (p = 0.0008). Heterogeneity among studies was high (I2 = 88%)

**Fig. 4:**
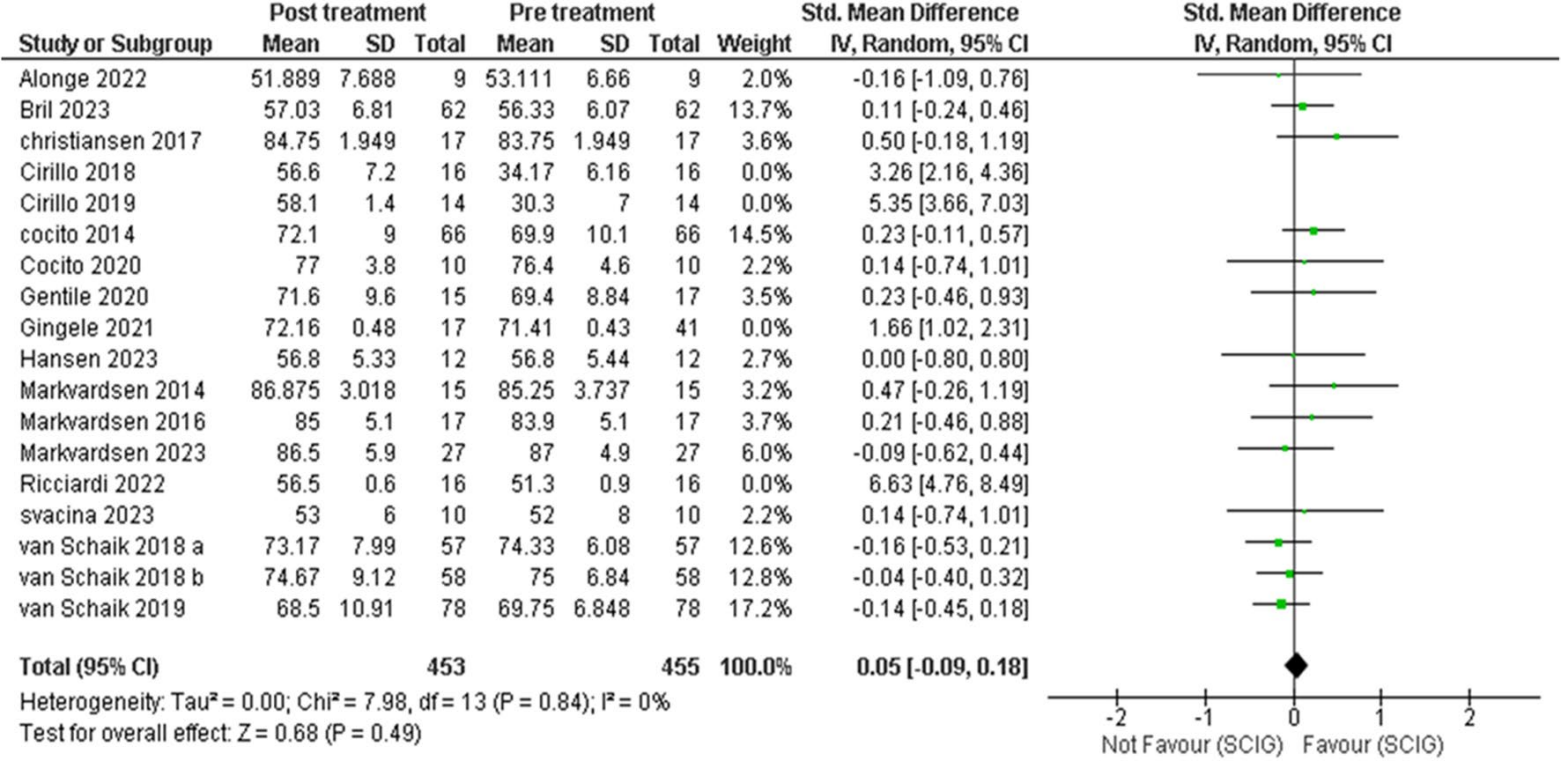
MRC after sensitivity analysis, heterogeneity was resolved after sensitivity analysis

**Fig. 5:**
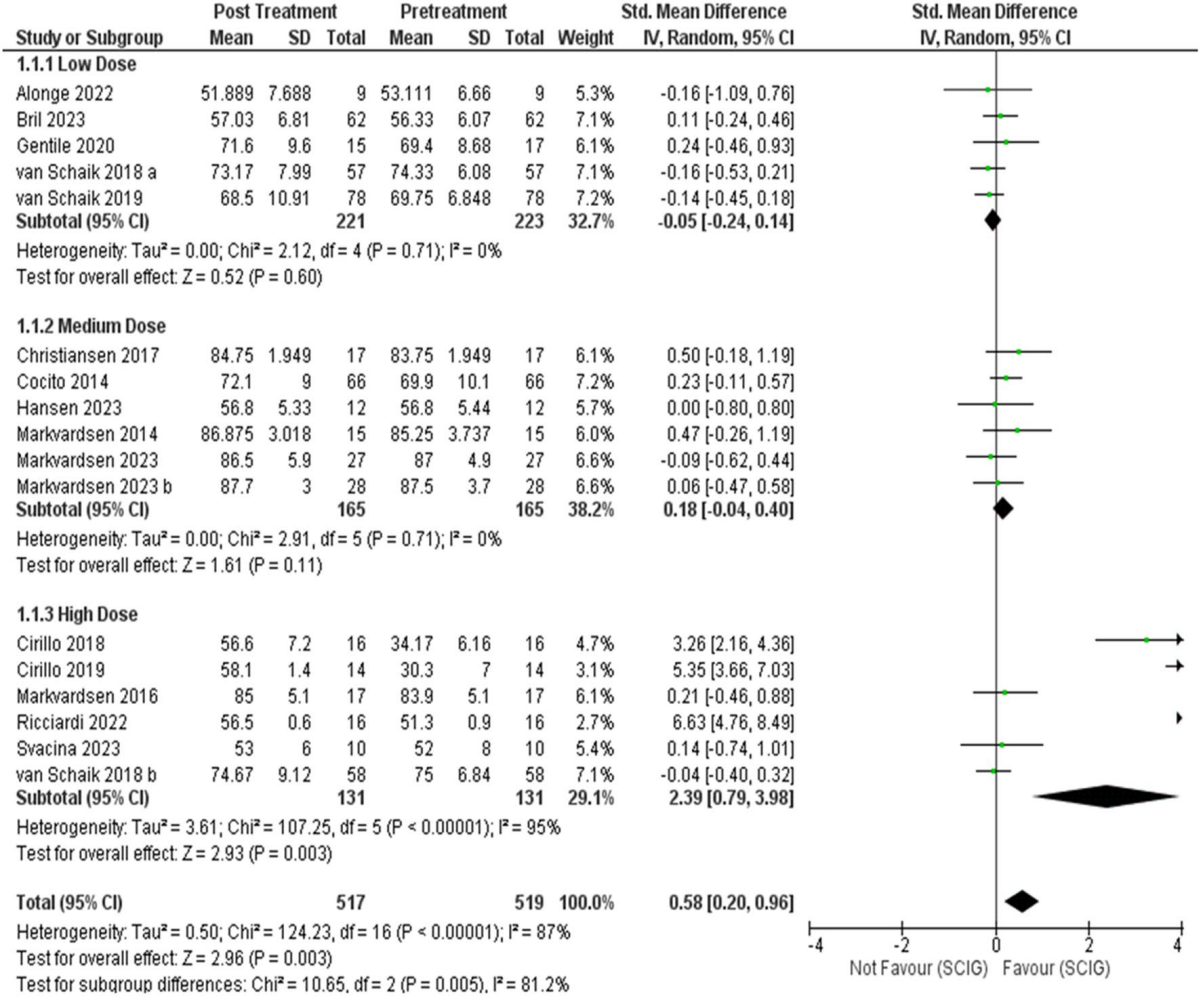
MRC Subgroup Analysis by Dose: Subgroups were divided by dose as low, medium, and high. The low dose subgroup shows no significant effect (SMD -0.05, 95% CI [-0.22, 0.14]). The medium dose subgroup shows a small but not statistically significant effect (SMD 0.18, 95% CI [-0.04, 0.40]). The high dose subgroup shows a significant effect (SMD 2.39, 95% CI [0.79, 3.98]) and high heterogeneity (I2 = 95%)

**Fig. 6:**
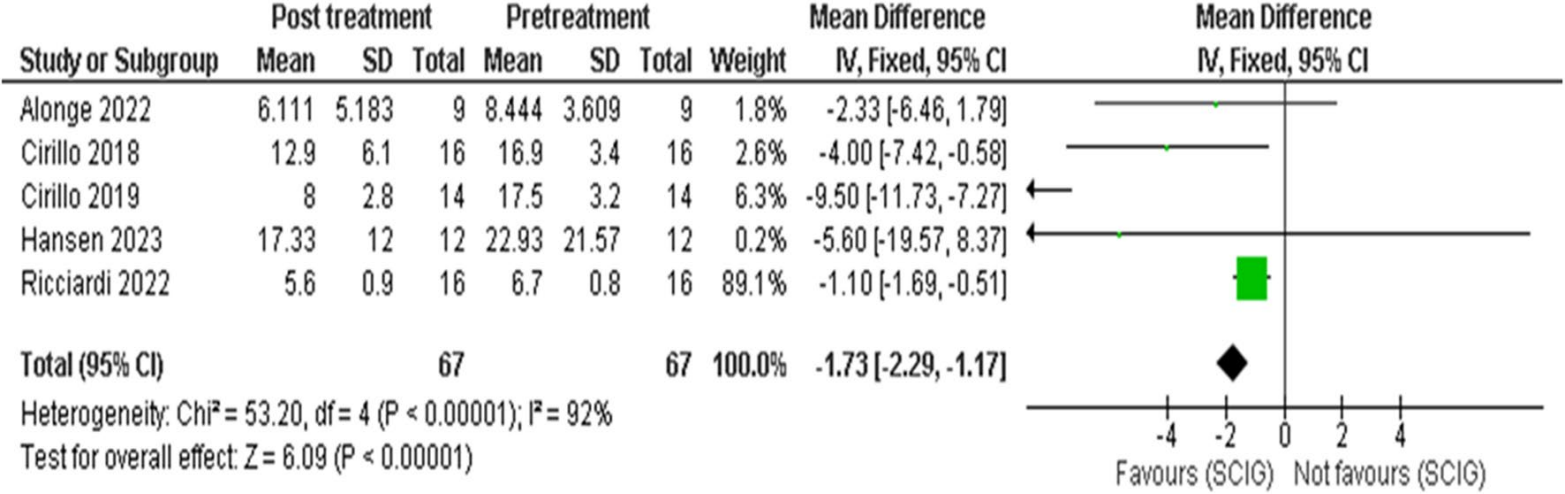
INCAT Sensory Score: Our analysis of 5 studies, including 67 participants, revealed a significant improvement in sensory function as measured by the INCAT Sensory Score. The pooled mean difference (MD) showed a reduction of 1.73 points (95% CI: -2.29 to -1.17), which was statistically significant (p < 0.00001). This improvement indicates enhanced sensory function following SCIG therapy, there was high heterogeneity (I2 = 92%) among the included studies

**Fig. 7:**
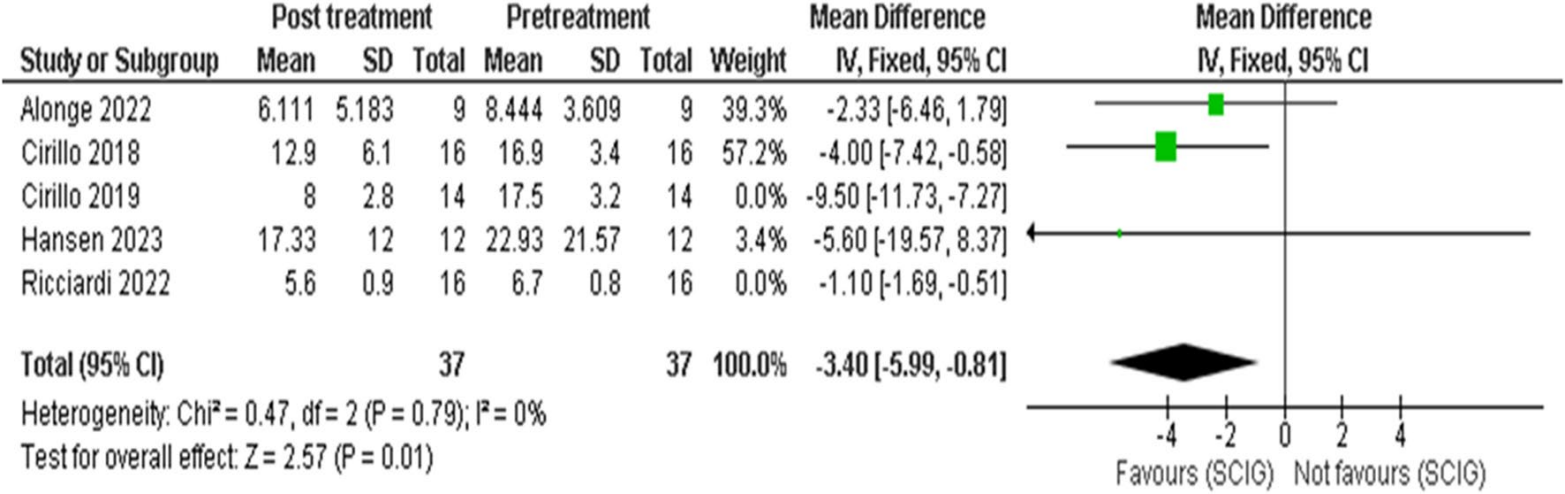
INCAT sensory after sensitivity analysis, heterogeneity was resolved in sensitivity analysis

**Fig. 8:**
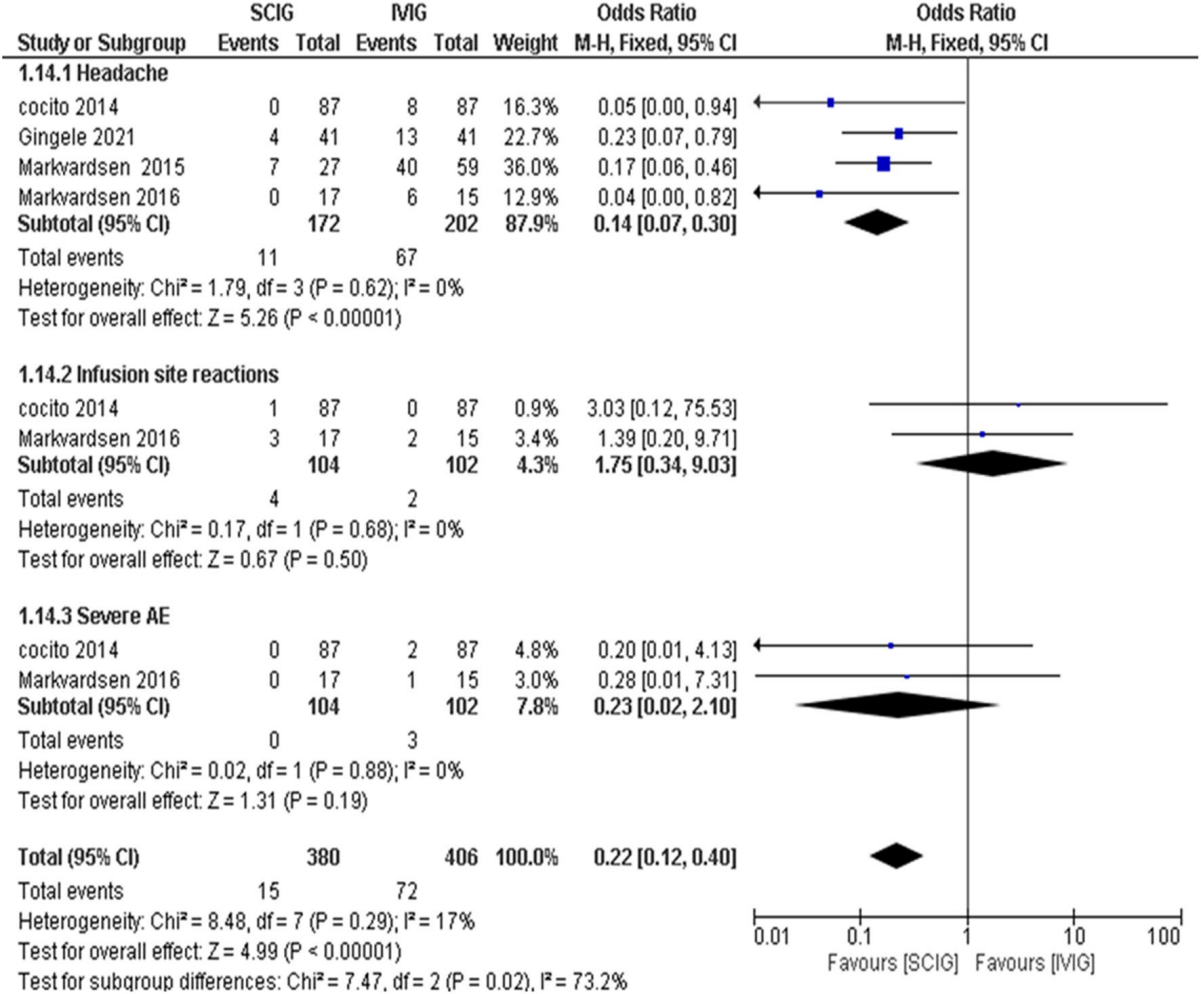
Side Effects Analysis: SCIG vs. IVIG: Overall Side Effects: From 4 studies (202 patients), side effects were less likely with SCIG, showing a significant pooled OR of 0.22 (95% CI: 0.12 to 0.40, p < 0.0001). Heterogeneity was insignificant (I2 = 17%) Headache: Analysis from 2 studies (202 patients) found significant difference in headache occurrence in SCIG group, OR = 0.14 (95% CI: 0.07 to 0.30, p < 0.0001) Infusion Site Reactions: Not significantly more common with SCIG in 2 studies (104 patients), with an OR of 1.75 (p = 0.50). No heterogeneity (I2 = 0%) Severe Side Effects: No significant differences were found in severe side effects (OR = 0.23, p = 0.19) across 2 studies (104 patients)

**Fig. 9:**
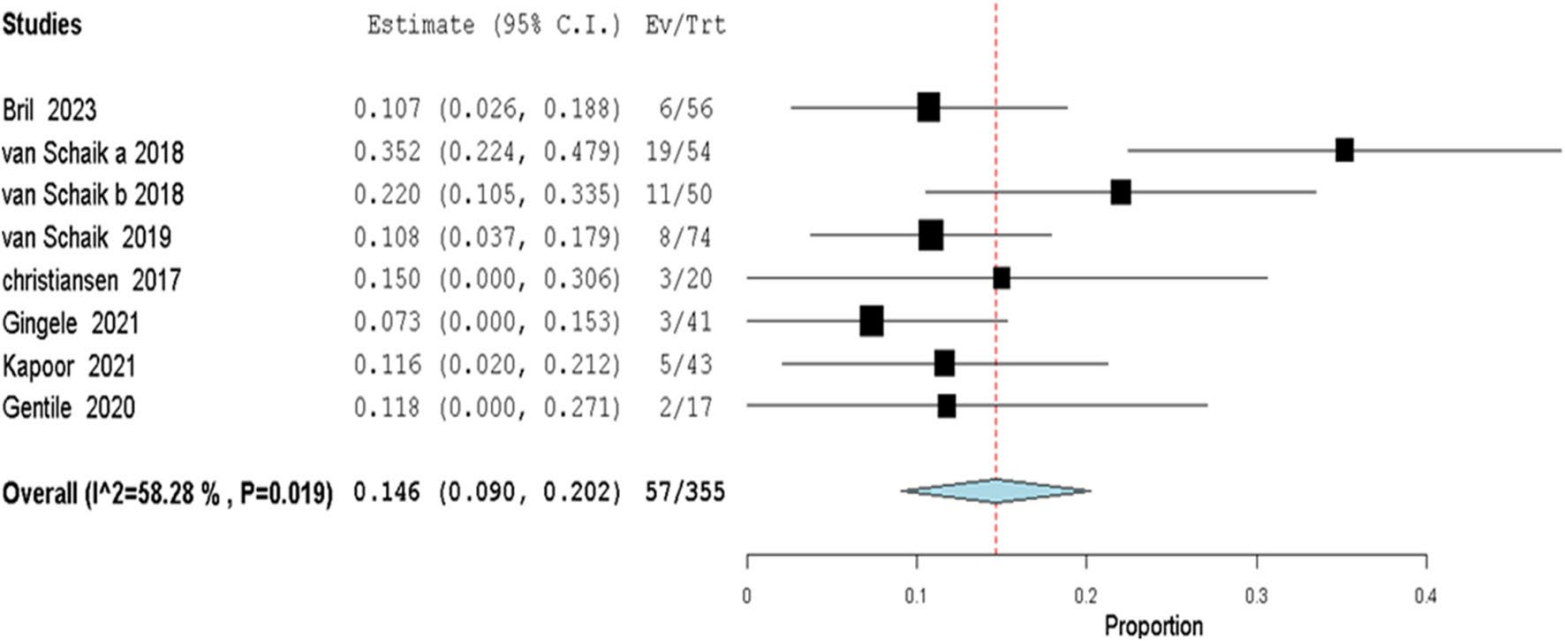
Relapse Rate Reduction: SCIG in CIDP: In CIDP patients treated with SCIG, a significant reduction in the relapse rate was observed. Analyzing data from 8 studies, the risk ratio was 0.146 (95% CI: 0.090 to 0.202), with a p-value < 0.001

### *Further Meta*-Analysis

The figures representing the meta-analyses of Quality of Life Measures, EQ-VAS, Timed Meter Walk Test, 9-HPT, Side Effects Analysis SCIG vs. Placebo, ODSS, IRODS, INCAT, Grip Strength, and IgG levels are provided in the supplementary materials.

## Discussion

This comprehensive Systematic Review and Meta-Analysis aimed to evaluate the efficacy, safety, and convenience of subcutaneous immunoglobulin (SCIG) in the maintenance treatment of Chronic Inflammatory Demyelinating Polyneuropathy (CIDP), covering a broad range of dimensions and outcomes, including relapse rate, overall function and disability, muscle strength, sensory function, serum IgG levels, quality of life, adverse events, patient treatment preferences, and economic implications.

To our knowledge, this can be considered the first systematic review or meta-analysis dedicated specifically to this particular topic, at least within accessible or peer-reviewed sources. The closest related meta-analysis is an older one by Racosta et al., comparing SCIG with intravenous immunoglobulin (IVIG) for chronic autoimmune neuropathies [60]; however, it is both less specific to our focus and less comprehensive, and it predates the main bulk of the current literature. Nonetheless, some of their findings align with ours in direction.

For this project, we compiled 50 clinical studies from 11 databases, 50 for the narrative syntheses, and 22 for the statistical syntheses.

### Summary of key findings and their integration with existing literature

Since SCIG is considered a maintenance therapy after stabilizing on IVIG [61], the primary objective is to avoid a recurrence and maintain or uphold neuromuscular function and overall quality of life [10]. Interestingly, our meta-analysis met the desired goal of SCIg treatment, showing that the relapse rate was relatively acceptable, and all the assessed efficacy outcomes were either maintained or improved.

In comparison to the conventional IVIG treatment, a previous 52-week open-label study found that IVIG administered as maintenance therapy had a relapse rate of 10.5% [62], similar to the results of high-dose SCIG treatment in the 48-week open-label PATH extension trial RR 10.8% [24]. Our results of 14.6% RR relate to these findings, indicating that both IVIG and SCIG might have comparable efficacy in terms of relapse rates.

In brief, the most important efficacy assessment outcome measures in CIDP include the INCATd scale and the R-ODS scale for disability, in addition to grip strength and the MRC sum score for muscle strength [63–65]. Analysis of these scores did not show any deterioration during the SCIg treatment compared to IVIG stabilized baseline, compatible with the prior Meta-analysis conclusion, noting that it assessed only the MRC sum score [60].

MRC's Initial meta-analysis results indicated a significant improvement in MRC scale scores post-SCIG treatment. Sensitivity analysis, however, revealed a non-significant effect, necessitating further examination through subgroup analysis. This analysis demonstrated a dose–response relationship, where higher SCIG doses correlated with significant improvements in MRC scores. However, a direct comparison between low and high doses in the PATH study did not reveal a discernible superiority of high-dose SCIg in MRC scores [26].

Furthermore, sensory impairment is considered one of the main hallmarks of CIDP and can be evaluated by the INCATss scale [66, 67]. Our findings demonstrated a statistically significant improvement in this scale, in favor of SCIg treatment even after sensitivity analysis, however, the studies that reported this outcome were not of high quality. For impaired functional mobility, no worsening was observed in the analysis of 9-HPT and timed meter walk test scores, indicating that patients preserved their functional mobility upon SCIG maintenance treatment.

The present analysis reveals that SCIG is associated with a 22% decreased risk of adverse events compared to IVIG. This observation is relatable to previous research, including Racosta's meta-analysis that noted a 28% reduction in the risk of moderate and systemic adverse events with SCIG versus IVIG [60]. Additionally, studies by Farmakidis et al. and Allen et al. support our findings, suggesting that SCIG leads to fewer adverse events relative to IVIg. This advantage is attributed to the slower absorption into the bloodstream with SCIG, which avoids the high peak levels of Immunoglobulin G (IgG) seen after IVIG administrations [8, 68].

Findings also showed that quality of life and health status remained stable after treatment with SCIG, which is consistent with IVIG treatments. This supports earlier research by Rajabally et al., which found that SCIg either improved or matched the outcomes of IVIg in treating CIDP [69]. It is worth noting that quality of life (QoL) is likely affected by the efficacy of immunoglobulin therapy [70], and since our analysis showed similar efficacy of SCIg and IVIg, stable QoL measures could be justified, especially when using general QoL scales like EQ-5D. Interestingly, two included studies that used a more IgG treatment oriented scale like LQI that considers many items related to patients' convenience, comfort, and independence according to the IgG route of administration [71], showed better QoL measures after SCIg. A previous pooled analysis of QoL in patients with Primary Immunodeficiency treated with Hizentra, an SCIG, may have reflected this by showing significant improvements in all LQI domain scores [72].

**Fig. 10:**
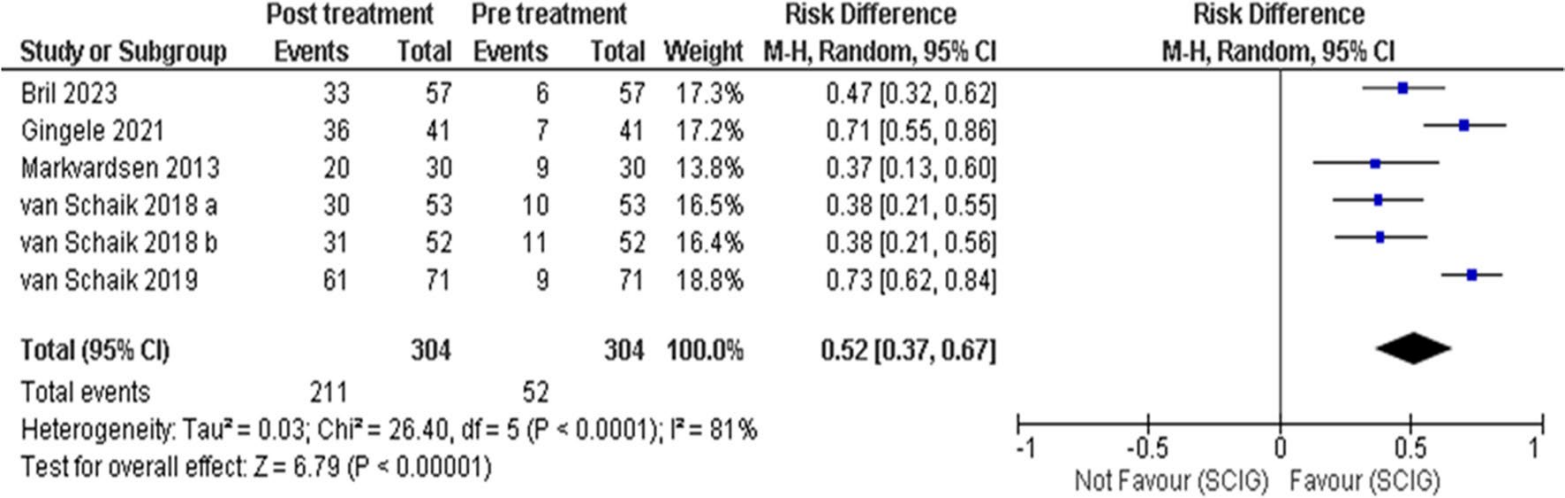
Treatment Preference: Analyzing data from 6 studies with 304 participants, there was a significant preference for SCIG over alternative treatments. The pooled Risk ratio (RR) was 0.52, with a 95% CI of 0.37 to 0.67, indicating a statistically significant preference (p < 0.0001). Heterogeneity was high (I2 = 81%)

Patients' treatment preferences, when analyzed, unanimously demonstrated a preference for SCIG across all studies. This includes, among others, probably the two largest studies in the field: PATH [26] and ADVANCE-CIDP1[10]. Studies that numerically reported a preference for SCIG over IVIG, depicted in Fig. 10, all have a mean age older than 55 years, with an overall weighted mean age of 57.

Unsurprisingly, this finding is in agreement with a previous systematic review on the burden of illness in CIDP by Querol et al., [73], as well as a literature review by Goyal et al.,[7]. Querol et al., highlights that 53–88% of patients favor SCIG over IVIG for its ease, flexibility, stability, and fewer side effects, also noting its time efficiency. Goyal et al., confirms a similar preference for SCIG among CIDP patients, attributing it to home administration convenience, lower volumes, fewer AEs, and fewer fluctuations.

An intriguing historical report notes that the initial application of immunoglobulin therapy for immune disorders commenced with subcutaneous administration in 1952, so it might after all come to what Wasserman elegantly phrased in a title of his, “from subcutaneous to intravenous infusions and back again” [74, 75].

The economic evaluations integrated into our analysis reveal that SCIG generally presents as a more cost-effective option, particularly when considering long-term treatment. These evaluations, conducted across various healthcare systems, indicate potential cost savings associated with SCIG, primarily attributed to reductions in hospitalization, administration costs, and the management of adverse events. These findings align with the broader trend towards home-based and patient-centered care models, suggesting that the adoption of SCIG should be considered within the specific context of each healthcare system.

### Strengths, limitations, and future research recommendations

This work offers a thorough and organized presentation and analysis of most available data, providing strong evidence supporting the use of SCIG for CIDP. Given CIDP's rarity, incorporating data from all available study designs is a notable strength, maximizing the use of limited information for a better understanding and management of this condition. However, the analysis encountered some limitations, especially in accessing specific raw data, despite reaching out to researchers. This brings to mind the broader issue of data transparency in scientific research and the call for future work to push for more open data sharing for richer analyses. Additionally, given the heterogeneity in certain outcomes and the partial reliance on predominantly indirect SCIG versus IVIG comparisons, a cautious interpretation of the findings is recommended.

Some kind of paradox emerged in Bril 2023, for example, when a seemingly synonymous or related group of constructs displayed some degree of divergence. Namely, in one study, the compound scale Treatment Satisfaction Questionnaire for Medication (TSQM) scores suggested some decrease in treatment satisfaction, yet the same patients from the same study reported simply an increased satisfaction and also separately a clear preference for SCIG treatment, while concurrently showing no significant change in health-related quality of life scales. This situation, observed in Bril 2023 and van Schaick 2018, highlights the complexity of interpreting patient-reported outcome measures (PROMs). Despite seemingly contradictory results, these instances reveal the nuanced nature of patient preferences and experiences in chronic disease management.

The analysis of economic outcomes, limited to countries with somewhat similar economic systems, further emphasizes the need for careful consideration when generalizing these results to different contexts.

Future research should focus on longitudinal studies comparing SCIG and IVIG as CIDP maintenance treatments to identify predictors of successful treatment transitions. Whenever possible, they should also refine inclusion criteria, grouping and outcome measures within individual studies to precisely differentiate between typical and atypical CIDP subtypes and other related conditions like MMN, enhancing inferential capacity and accessibility. Expanding economic impact studies of these therapies across diverse economies is also essential.

It is also advisable for some future research lines and guidelines to consider giving more weight to specialized physiotherapy for CIDP maintenance. Although not yet well-documented in the literature and not our focus here, practical experience suggests it could be of pivotal importance in sustaining remission and perhaps could be considered as an add-on to SCIG for an increased, additive, or even synergistic benefit.

Furthermore, exploring the Facilitated SCIG (FSCIG), which holds the potential to combine the main advantages of SCIG and IVIG, presents a promising research direction that could significantly impact CIDP treatment strategies. The increasing capability of SCIG to administer larger doses of immunoglobulins could indicate its potential for use in CIDP induction therapy in the future [76].

## Conclusion

The findings suggest that SCIG for CIDP maintenance not only provides a more feasible alternative, with economic evaluations showing considerable cost reductions over time and patient preference for SCIG being pronounced, but may also deliver comparable or superior health outcomes.

A patient-focused strategy in handling CIDP, guided by robust evidence and economic considerations, aiming for optimal patient outcomes and satisfaction, shall highlight the importance of SCIG for those suited to it.

Ongoing research lines on formulations, techniques, and direct comparative studies are critical to further illuminate, enhance, and expand SCIG's role in treatment, ensuring medical practices are equipped with optimal tools, informed by comprehensive evidence, are cost-effective, and probably most importantly, aligned with patient preferences and convenience.

## Supplementary Information

Below is the link to the electronic supplementary material.

Supplementary file1 (XLSX 1569 KB)

## Data Availability

Provided in the supplementary material.
